# *NSUN2* mediates *SALL2* m5C methylation to inhibit ferroptosis and promote breast cancer progression

**DOI:** 10.1080/15384047.2026.2695501

**Published:** 2026-06-30

**Authors:** Jiawei Li, Min Li, Jianran Guo, Dongbao Li, Yumin Yao, Wei Zhang, Chuanyou Cui, Bo Fu

**Affiliations:** a Department of Precision Biomedical Key Laboratory, Liaocheng People’s Hospital, Liaocheng Hospital Affiliated to Shandong First Medical University, Shandong Provincial Key Medical and Health Laboratory of Precision Medicine for Aging Intervention and Active Health, Liaocheng, People's Republic of China; b Liaocheng Key Laboratory for Precision Diagnosis and Treatment of Malignant Tumors, Liaocheng People’s Hospital, Liaocheng Hospital Affiliated to Shandong First Medical University, Liaocheng, People's Republic of China; c Department of Breast Surgery, Liaocheng People’s Hospital, Liaocheng Hospital Affiliated to Shandong First Medical University, Liaocheng, People's Republic of China; d Liaocheng Key Laboratory of Breast Diseases, Liaocheng People’s Hospital, Liaocheng Hospital Affiliated to Shandong First Medical University, Liaocheng, People's Republic of China; e Medical Integration and Practice Center, Shandong University, Jinan, People's Republic of China

**Keywords:** *SALL2*, breast cancer, m5C methylation, *NSUN2*, ferroptosis

## Abstract

**Background:**

Due to the heterogeneity of breast cancer (BRCA) and the limited therapeutic efficacy in specific molecular subtypes, identifying new and effective therapeutic targets remains an urgent clinical need. The transcription factor *SALL2* has been shown to play distinct roles in tumorigenesis and progression, but its specific role in BRCA remains unclear. Therefore, this study investigates the effects of *SALL2* on BRCA progression.

**Methods:**

*SALL2* expression in the TCGA and clinical BRCA tissue samples was analyzed. Cell and animal models were established to validate the effects of *SALL2* on BRCA cell proliferation, migration, and tumorigenicity. Molecular experiments were performed to investigate the association between NSUN2 and SALL2 in BRCA and cellular ferroptosis regulation by SALL2.

**Results:**

*SALL2* is significantly overexpressed in BRCA tissues and cell lines, knocking down *SALL2* reduces BRCA cell growth, migration, and tumorigenesis *in vitro* and *in vivo* models. The m5C methyltransferase NSUN2, together with the reader protein YBX1, modifies *SALL2* mRNA with m5C, which stabilizes the transcript. *SALL2* then acts as a transcriptional repressor by binding directly to the promoter region of *ACSL4* and inhibiting its transcriptional activity. Suppression of *SALL2* activates ferroptotic cell death by lowering the antioxidant defences of GPX4/SLC7A11 and increasing the expression of *ACSL4* and *COX2*. Ferroptosis inhibitors can reverse the growth-inhibiting effects caused by *SALL2* depletion.

**Conclusion:**

In BRCA, *SALL2* is precisely regulated by m5C RNA methylation modification, further promoting cancer progression by inhibiting cell ferroptosis, suggesting its potential as a prognostic biomarker and a target for therapy.

## 1. Introduction

Breast cancer (BRCA) is the most common female malignancy, and accounted for 25% of all female cancer cases in 2022. Despite variations in patient age and geographical location, the overall incidence of BRCA has risen in many countries over the past decade, placing a heavy burden on women’s health worldwide.^1^ ^,^ ^2^ Despite significant progress in treatments like surgery, chemotherapy, radiotherapy, endocrine therapy, and targeted molecular therapies, many patients, especially those with aggressive or resistant forms, face unsatisfactory outcomes.^3^ ^,^ ^4^ While immunotherapy has shown promise in some cancers, its efficacy in BRCA-mutated cases remains limited to a small subset.^5^ ^,^ ^6^ Therefore, understanding the molecular mechanisms underlying BRCA development and progression and identifying new, effective therapeutic targets are urgent priorities in BRCA research.

Breast cancer shows significant molecular and phenotypic diversity, mediated by complex genetic and epigenetic regulatory networks that control tumor development, progression, and treatment response. The Spalt-like (SALL) protein family consists of conserved C2H2 zinc-finger transcription factors vital for embryonic development, cell fate decisions, and tissue formation. Current evidence suggests that abnormal expression, structural changes, or irregular isoform production of SALL proteins are common in various cancers, often resulting from genetic or epigenetic modifications. These abnormalities promote tumor formation by influencing key cellular functions, including proliferation, apoptosis, differentiation, and migration.^7^ SALL2, a key member of the SALL protein family, participates in biological functions including cellular differentiation, development, and adhesion.^8^ Its roles in cancer are context-dependent, showing both tumor-suppressing and tumor-promoting activities influenced by tumor type, cellular environment, and microenvironmental factors. For example, *SALL2* has been found to inhibit ovarian cancer progression, while showing oncogenic features in glioblastoma. In BRCA, there is growing evidence linking *SALL2* expression to chemotherapy resistance.^9^ ^,^ ^10^ Additionally, a study by Sandeep Sisodiya et al. reported both subtype-dependent expression and mutations in *SALL2.* ^11^ However, its exact biological role, regulatory pathways, and impact on BRCA development are not well understood and need further research.^12^ ^,^ ^13^

Epigenetic regulation plays a pivotal role in BRCA development, influencing malignant transformation, invasion, metastasis, and therapeutic resistance.^14^ ^,^ ^15^ Among epigenetic mechanisms, RNA methylation has emerged as a key regulator of gene expression at the post-transcriptional level. In particular, the 5-methylcytosine (m5C) RNA modification has attracted increasing attention due to its involvement in RNA metabolism, transcript stability, and translational efficiency.^16^ ^,^ ^17^ The m5C modification is orchestrated by the synergistic activity of methyltransferases (writers), demethylases (erasers), and m5C-binding proteins (readers). The NSUN family of methyltransferases (NSUN1-NSUN7) contains a conserved catalytic domain (approximately 270 amino acids) and an S-adenosylmethionine-binding site that allows the deposition of m5C marks on RNA substrates. Among these enzymes, NSUN2 has been identified as a main mediator of mRNA m5C methylation due to its broad substrate specificity.^18-21^ Dysregulation of NSUN2 has been closely associated with malignant progression and drug resistance in multiple tumor types through m5C-dependent modulation of oncogenic transcripts.^22^ ^,^ ^23^ Y-box binding protein 1 (YBX1), a well-characterized m5C reader, selectively recognizes m5C-modified mRNAs, enhances their stability, and promotes protein translation.^24^ Studies indicate that the NSUN2-YBX1-m5C axis plays a crucial role in maintaining oncogenic signaling pathways and driving tumor progression.^25-27^

Intracellular ion homeostasis plays a key role in the onset and progression of malignancy, with both ion excess and deficiency affecting cellular function.^28-30^ Ferroptosis is a distinct, non-apoptotic form of regulated cell death induced by iron-dependent lipid peroxidation. The molecular mechanism of ferroptosis has been progressively elucidated. The biosynthesis and peroxidation of PUFA-PLs highlight ferroptotic cell death, with enzymes such as ACSL4 and ALOX15 serving as key catalysts of lipid peroxidation. In comparison, the glutathione (GSH)-glutathione peroxidase 4 (GPX4) axis serves as the primary defense against ferroptosis. SLC7A11 mediates cystine uptake for GSH synthesis, while GPX4 uses GSH to detoxify lipid peroxides. Disruption of this protective axis, through SLC7A11 suppression or GPX4 inactivation, inevitably leads to ferroptotic cell death.^29^ ^,^ ^31^ ^,^ ^32^ Recent studies have highlighted the relevance of ferroptosis in BRCA pathophysiology. For example, CD36 inhibits the progression of TNBC by promoting ferroptosis,^33^ while crVDAC3 confers resistance to breast cancer by alleviating ferroptotic stress in tumor cells.^34^ In addition, a study by Gong et al. found that inhibiting ferroptosis also exacerbates the vicious cycle of bone metastasis in breast tumors.^35^ These findings show the crucial involvement of ferroptosis in BRCA progression and therapeutic response.

This study examined the biological role of *SALL2* in BRCA progression using both *in vitro* and *in vivo* models. The upstream epigenetic mechanisms responsible for aberrant *SALL2* expression were elucidated, with particular focus on m5C RNA methylation-mediated regulation. Moreover, the potential role of *SALL2* in modulating ferroptosis signaling pathways during BRCA progression was explored. These findings provide crucial mechanistic understanding into BRCA pathogenesis and identify promising molecular targets for prognostic evaluation and therapeutic intervention.

## 2. Results

### 2.1. *SALL2* is overexpressed in breast cancer and correlates with poor patient prognosis

**Figure 1.**
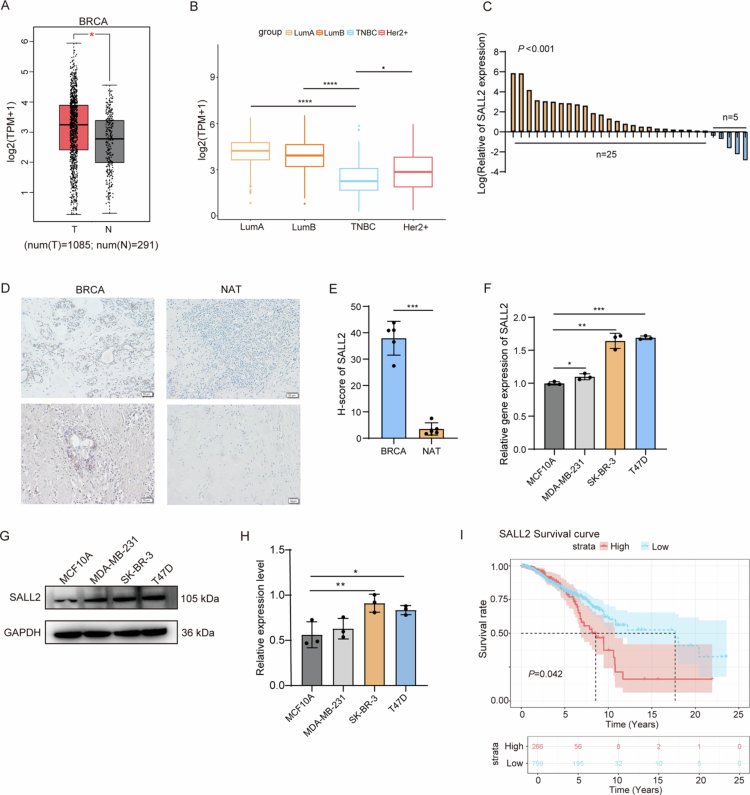
SALL2 is highly expressed in breast cancer. A. Differences in *SALL2* expression between normal and tumor tissues in breast cancer (Tumor, *n* = 1085. Normal, *n* = 291). B. *SALL2* expression across breast cancer subtypes. C. RT-qPCR detection of *SALL2* expression levels in breast cancer and normal adjacebt samples. D,E. Immunohistochemical staining for SALL2 in BRCA tumor tissues and paired normal adjacebt tissues. (D) Representative IHC images. Scale bars, 50 μm. (E) Quantification of H-scores. F–H. Validation of *SALL2* expression in normal breast epithelial cells and multiple BRCA cells via RT-qPCR (F) and Western blotting (G, H). I. Correlation between SALL2 expression and survival duration in breast cancer patients. (*p* < 0.0001, “****”, *p* < 0.001, “***”, *p* < 0.01, “**”, *p* < 0.05, “*”).

To characterize the expression pattern of *SALL2* in BRCA, transcriptomic data from the TCGA-BRCA dataset were analyzed and compared with normal breast tissue samples obtained from the GTEx database. The analysis showed that *SALL2* expression was significantly increased in BRCA samples relative to normal tissues (tumor, *n* = 1085; normal, *n* = 291; *p* < 0.05) (Figure 1A). Stratification according to molecular subtype further revealed heterogeneity in SALL2 expression, with the highest levels observed in Luminal A tumors, followed by Luminal B, HER2-positive tumors, and the lowest expression detected in triple-negative breast cancer (TNBC) (Figure 1B).

To confirm these *in silico* findings, 30 matched pairs of breast carcinoma tissues and normal adjacent breast tissues were examined. RT-qPCR analysis confirmed a significant elevation of *SALL2* mRNA levels in tumor samples compared with respective normal adjacent tissues (Figure 1C), consistent with the bioinformatic results. Similarly, immunohistochemical analysis showed stronger SALL2 protein staining in BRCA tissues compared to normal adjacent tissues (Figure 1D, E). At the cellular level, RT-qPCR showed higher expression of *SALL2* in multiple BRCA cell lines (MDA-MB-231, SK-BR-3, and T47D) than in the normal mammary epithelial cell line MCF10A (Figure 1 F), and this finding was further validated by Western blotting (Figure 1G, H). To observe the prognostic relevance of *SALL2*, survival analysis was conducted. Kaplan-Meier survival curves indicated that patients with *SALL2* overexpression had significantly reduced overall survival compared with those with lower SALL2 expression (Figure 1I). These data indicate that *SALL2* is frequently overexpressed in BRCA and is associated with an adverse prognosis.

### 2.2. *SALL2* promotes breast cancer cell growth, migration, and tumorigenicity

**Figure 2.**
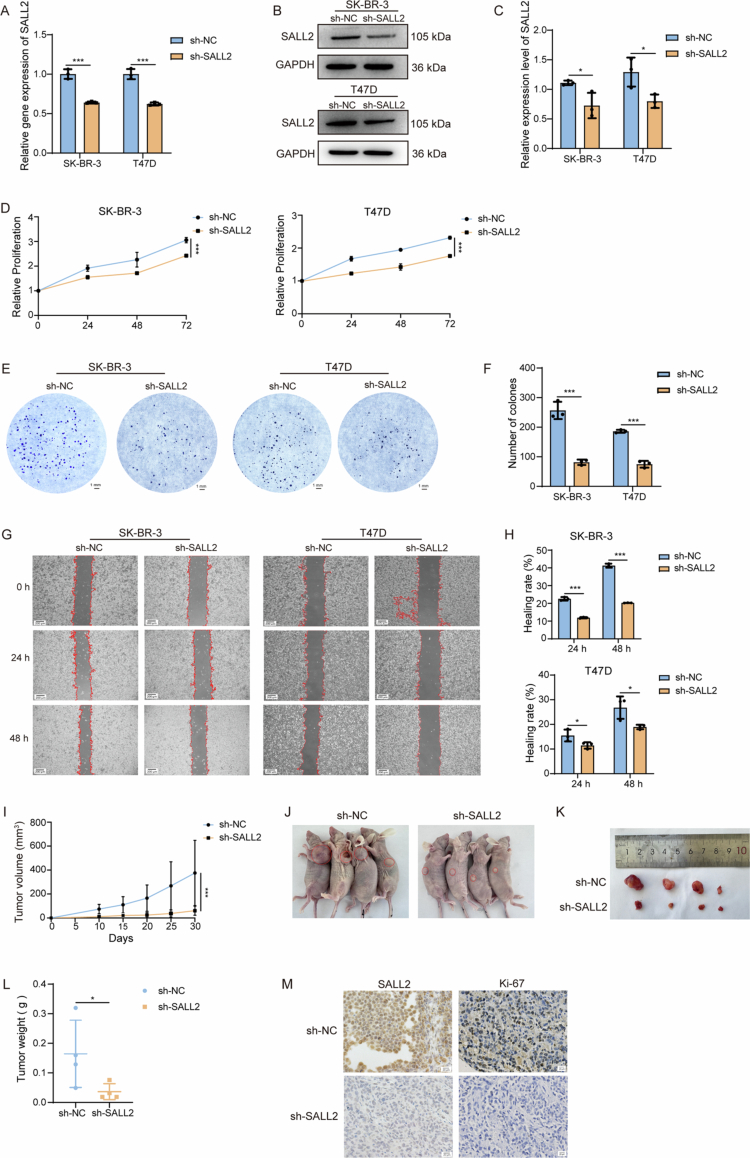
*SALL2* promotes proliferation and migration of breast cancer cells. A−C. RT-qPCR (A) and Western blot (B, C) detection of SALL2 knockdown efficiency. D−F. CCK-8 (D) and colony formation (E, F) assays validate *SALL2*'s effect on breast cancer cell proliferation *in vitro*. Scale bars, 1mm. G,H. Wound healing assay verifying *SALL2*'s effect on breast cancer cell migration. Scale bars, 200 μm. I−L. *SALL2* affects breast cancer cell proliferation *in vivo*. (I). Subcutaneous tumor growth curve. (J). Subcutaneous tumor images in mice. (K). Excisional tumor images after removal. (L). Excisional tumor weight. M. IHC detection of SALL2 and Ki-67 expression in tumors from both groups. Scale bars, 20 μm. (*p* < 0.001, “***”, *p* < 0.01, “**”, *p* < 0.05, “*”).

To evaluate the functional role of *SALL2* in BRCA progression, stable *SALL2* knockdown models were developed in SK-BR-3 and T47D cell lines using lentiviral shRNA constructs (sh-SALL2), with cells transduced with a non-targeting shRNA serving as controls (sh-NC). Quantitative PCR and Western blot analyzes confirmed a robust reduction in SALL2 expression at both the transcript and protein levels in sh-SALL2 cells compared with control cells (Figure 2A−C). Cell proliferation assays using the CCK-8 assay showed that depletion of *SALL2* significantly reduced the proliferative capacity of both BRCA cell lines (Figure 2D). Consistent with this, colony formation assays showed a significant decrease in the number and size of colonies formed by *SALL2*-silenced cells compared with controls (Figure 2E, F). Furthermore, wound healing assays showed that the migratory ability of BRCA cells was substantially attenuated after *SALL2* knockdown (Figure 2G, H). These *in vitro* findings indicate that reduced *SALL2* expression compromises BRCA cell proliferation and migration.

To further confirm these observations *in vivo*, subcutaneous xenograft models were developed in nude mice. Tumors derived from sh-SALL2 cells grew significantly more slowly than those formed by control cells (Figure 2I). At the experimental endpoint, both tumor volume (Figure 2J, K) and tumor weight (Figure 2L) were significantly lower in the *SALL2* knockdown group. Immunohistochemical analysis of excised tumor tissues revealed a significant reduction in the proportion of Ki67-positive cells in the sh-SALL2 group, indicating decreased proliferative activity (Figure 2M). These *in vitro* and *in vivo* results consistently demonstrate that *SALL2* enhances BRCA cell proliferation, migration, and tumorigenic potential, supporting its oncogenic role in BRCA progression.

### 2.3. *NSUN2* upregulates *SALL2* expression through m5C methylation of SALL2 mRNA

**Figure 3.**
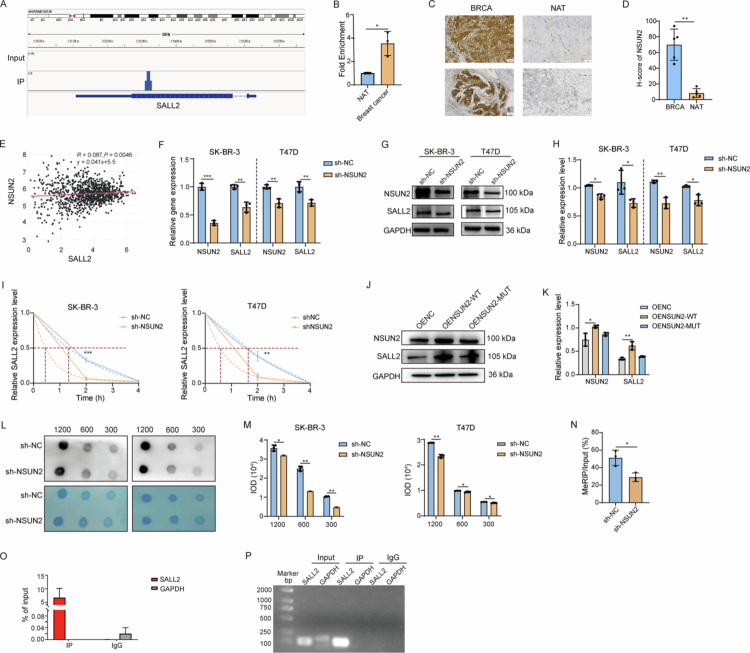
*NSUN2* upregulates *SALL2* expression by mediating its mRNA m5C methylation modification. A. SALL2 mRNA exhibits high m5C methylation modification in breast cancer. B. MeRIP-qPCR detection of SALL2 m5C methylation levels in breast cancer and normal adjacent tissues. C,D. IHC staining for NSUN2 in BRCA tissues and paired normal adjacebt tissues. (C) Representative IHC images. Scale bar, 100 μm. (D) Quantification of H-scores. E. Correlation analysis between *NSUN2* and *SALL2* in breast cancer. F−H. RT-qPCR (F) and Western blot (G, H) assays for SALL2 expression following NSUN2 knockdown. **I.** Decay assays for SALL2 RNA stability after NSUN2 knockdown. J,K. Western blot analysis of SALL2 expression levels following NSUN2 overexpression. L,M. RNA dot blot analysis of m5C methylation levels in NSUN2-knockdown breast cancer cells. N. MeRIP-qPCR assay of m5C methylation levels of SALL2 following NSUN2 knockdown. O,P. RIP-qPCR (O) and agarose gel electrophoresis (P) validate the interaction between SALL2 and YBX1. (*p* < 0.001, “***”, *p* < 0.01, “**”, *p* < 0.05, “*”).

Our previous research revealed distinct m5C methylation patterns in the transcriptome of breast cancer tissues.^36^ Among these differentially methylated transcripts, SALL2 showed elevated m5C methylation levels (Figure 3A). The results demonstrated that m5C enrichment on *SALL2* mRNA was elevated by approximately 3.5-fold in tumor tissues compared to normal adjacent tissues (Figure 3B), suggesting that abnormal post-transcriptional methylation may enhance its expression. Since NSUN2 is a key methyltransferase for mRNA m5C addition,^23^ its role in regulating *SALL2* was examined. Immunohistochemical staining demonstrated elevated NSUN2 levels in BRCA tissues relative to normal adjacent tissues (Figure 3C, D). Moreover, pan-cancer analysis of TCGA data revealed a significant positive correlation between *NSUN2* and *SALL2* expression in BRCA samples (Figure 3E; R = 0.087, *p* = 0.0046). To directly observe the regulatory effect of *NSUN2* on *SALL2*, *NSUN2* expression was silenced in SK-BR-3 and T47D cells. Reducing NSUN2 led to a substantial decrease in SALL2 mRNA and protein levels (Figure 3F−H). RNA decay analyzes further showed that depletion of *NSUN2* significantly shortened the half-life of *SALL2* transcripts (SK-BR-3: 1.714 h *vs.* 0.443 h; T47D: 1.895 h *vs.* 0.579 h), indicating compromised mRNA stability (Figure 3I). Rescue experiments demonstrated that ectopic expression of wild-type NSUN2 increased *SALL2* expression, whereas introduction of a catalytically inactive NSUN2 mutant (C271A/C321A) failed to produce a similar effect (Figure 3J, K), confirming that NSUN2-mediated regulation of *SALL2* depends on its enzymatic activity. To determine whether *NSUN2* exerts its regulatory function through m5C modification of *SALL2* mRNA, RNA dot blot analysis was performed and revealed a global reduction in cellular m5C levels after *NSUN2* knockdown (Figure 3L, M). Consistent with this, targeted MeRIP-qPCR analysis showed a significant decrease in m5C enrichment on *SALL2* transcripts in NSUN2-deficient cells (Figure 3N).

Since m5C-modified RNAs typically require recognition by specific reader proteins for stability,^37^ ^,^ ^38^ the involvement of YBX1 was examined. RIP-qPCR assays confirmed that YBX1 directly associates with *SALL2* mRNA (Figure 3O), and agarose gel electrophoresis further indicated that this interaction was dependent on intact m5C modification sites (Figure 3P). Therefore, these findings suggest that NSUN2 mediates m5C methylation of *SALL2* mRNA via its catalytic domain (C271A/C321A), which promotes YBX1 binding, stabilizes the transcript, and increases *SALL2* expression.

### 2.4. Effect of *NSUN2* on breast cancer cells

**Figure 4.**
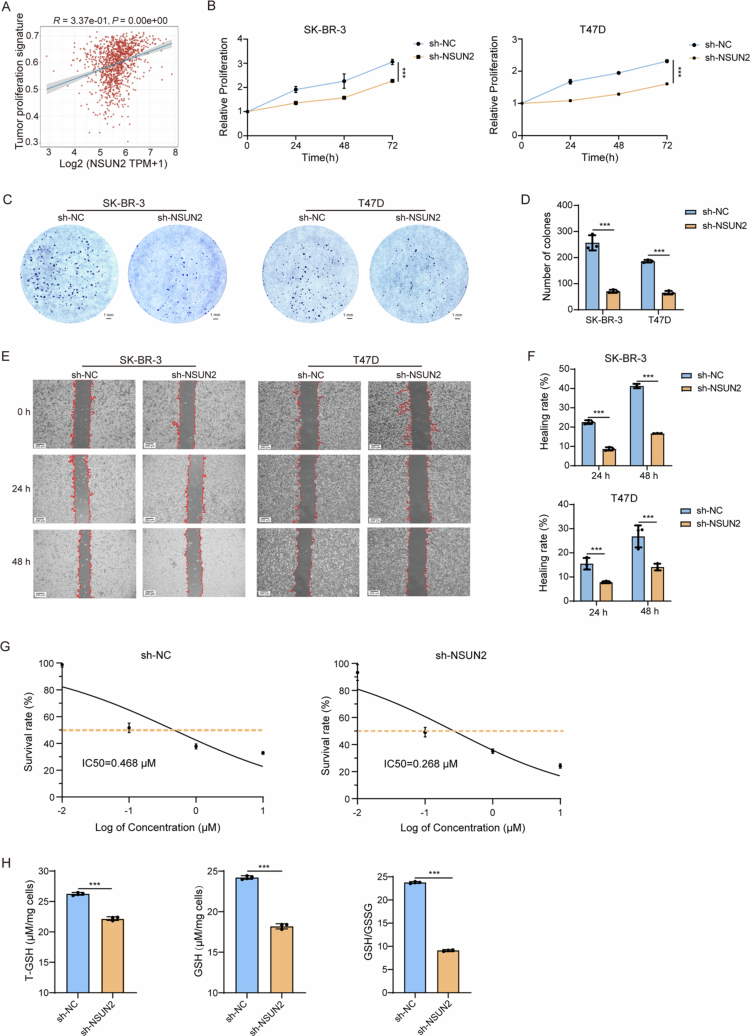
*NSUN2* affects the proliferation and migration of breast cancer cells. A. Correlation analysis between *NSUN2* and tumor proliferation signature. B−D. CCK-8 assay (B) and colony formation assay (C, D) validate the effect of *NSUN2* on breast cancer cell proliferation. Scale bars, 1 mm. E,F. Wound healing assay verifies the effect of *NSUN2* on breast cancer cell migration. Scale bars, 200 μm. G. Assessment of paclitaxel sensitivity in BRCA cells upon NSUN2 knockdown. H. Detection of T-GSH, GSH levels and GSH/GSSG ratio in BRCA cells following NSUN2 knockdown. (*p* < 0.001, “***”, *p* < 0.01, “**”, *p* < 0.05, “*”).

To evaluate the role of NSUN2 in BRCA progression, TCGA-BRCA data were observed through bioinformatics analysis. Spearman correlation analysis revealed a strong positive association between *NSUN2* expression and the enrichment of proliferation-related gene signatures (Figure 4A; R = 0.337, *p* < 0.001), indicating that elevated NSUN2 levels may contribute to enhanced tumor cell proliferation.

To experimentally verify this, *NSUN2* was silenced in SK-BR-3 and T47D cells using shRNA-mediated knockdown (sh-NSUN2), with sh-NC as the control group. Functional assays showed that *NSUN2* knockdown significantly reduced cell proliferation, as measured by CCK-8 assays (Figure 4B). Similarly, long-term clonogenic potential was substantially decreased in *NSUN2*-deficient cells, as evidenced by colony formation assays (Figure 4C, D). Furthermore, wound-healing assays revealed that NSUN2 knockdown significantly reduced BRCA cell migration (Figure 4E, F). Thus, these results indicate that NSUN2 enhances both the proliferative and migratory abilities of BRCA cells *in vitro*, confirming the bioinformatics analysis and supporting its pro-tumorigenic role. The effects of NSUN2 on BRCA cell sensitivity to paclitaxel, an anticancer drug widely used for BRCA treatment, were investigated. The results showed that NSUN2 knockdownsignificantly enhanced the cytotoxicity of paclitaxel against breast cancer cells, as reflected by a reduced IC_50_ (sh-NC *vs.* sh-NSUN2: 0.468 *vs.* 0.268, *p* < 0.001) (Figure 4G). These findings suggest that *NSUN2* is a potential regulator of drug sensitivity in BRCA cells.

Measurement of intracellular glutathione levels revealed that the concentrations of both T-GSH and GSH were significantly decreased in the sh-NSUN2 group compared with the control group. The GSH/GSSG ratio was also markedly reduced (Figure 4H). These data demonstrate that NSUN2 silencing accelerates glutathione consumption in BRCA cells, which is a hallmark of ferroptosis.^29^

### 2.5. *SALL2* deficiency inhibits the proliferation of breast cancer cells by inducing ferroptosis

**Figure 5.**
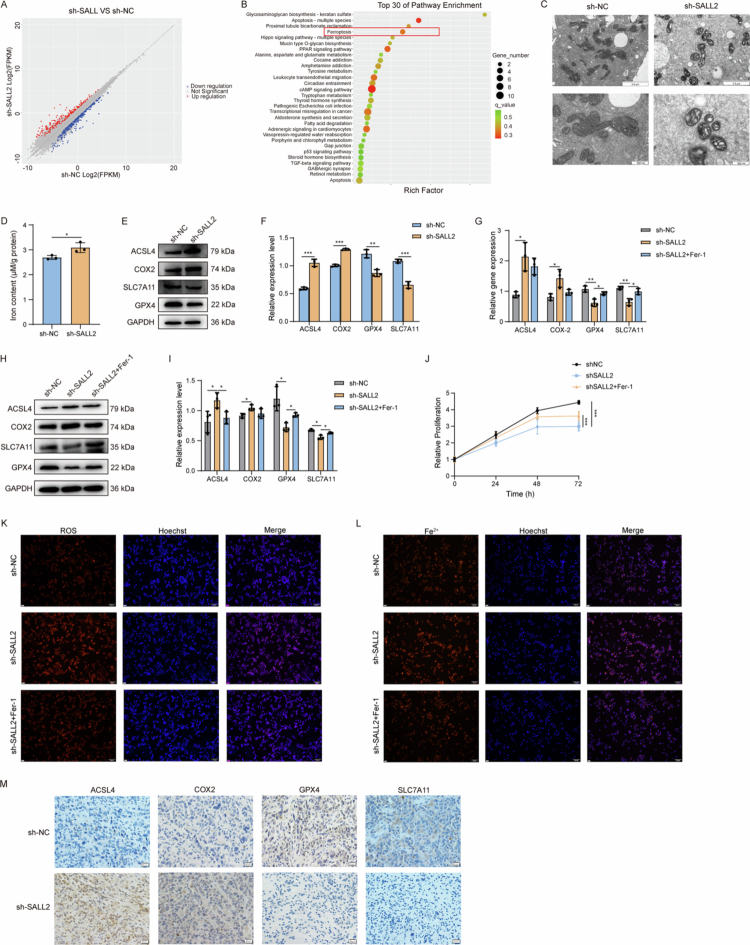
*SALL2* deficiency induces ferroptosis in breast cancer cells. A. Scatter plot of differentially expressed genes in sh-NC and sh-SALL2 cell groups. B. KEGG pathway analysis of differentially expressed genes. C. Observe and analyze the morphological differences in mitochondria between the two groups of cells using transmission electron microscopy. Scale bars: upper panels, 2 μm; lower panels, 500 nm. D. The iron ion concentration in sh-NC and sh-SALL2 cells. E,F. Western blot analysis of the effects of SALL2 knockdown on the expression of ferroptosis-related molecules. G. RT-qPCR analysis of the effects of SALL2 knockdown and SALL2 knockdown combined with ferroptosis inhibitor (Fer-1) treatment on ferroptosis-related factors. H,I. Western blot analysis of the effects of SALL2 knockdown and Fer-1 treatment on the expression of ferroptosis-related molecules. J. CCK-8 assay of the effects of SALL2 knockdown and Fer-1 treatment on cell proliferation. K,L. Fluorescent staining for intracellular ROS (K) and intracellular Fe^2+^ (L). Scale bars, 100 μm. M. IHC analysis of the expression of ferroptosis-related molecules in subcutaneous tumor tissues. Scale bars, 20 μm. (*p* < 0.001,“***”, *p* < 0.01,“**”, *p* < 0.05, “*”).

To investigate the downstream mechanisms by which *SALL2* influences BRCA progression, RNA sequencing (RNA-seq) was performed on SK-BR-3 cells with *SALL2* knockdown (sh-SALL2) and control cells (sh-NC). Using |log2 Fold Change| ≥ 1 and *p* ≤ 0.05 as criteria, 582 DEGs were identified, including 266 upregulated and 316 downregulated genes (Figure 5A). KEGG pathway enrichment analysis indicated that these DEGs were predominantly associated with ferroptosis and related signaling pathways (Figure 5B), suggesting that *SALL2* may modulate ferroptotic processes to influence BRCA cell fate. To validate this prediction, ultrastructural analysis of cells was performed using TEM. Knockdown of *SALL2* resulted in mitochondria exhibiting classical ferroptotic features, including reduced size, loss of cristae, and increased membrane density^39^ (Figure 5C). Next, intracellular iron levels were quantified, revealing a significant increase in iron ion concentration in sh-SALL2 cells compared with controls (Figure 5D), indicating disrupted iron homeostasis. At the molecular level, key ferroptosis-associated regulators were examined. Western blot and RT-qPCR analyzes showed that *SALL2* depletion significantly downregulated the expression of antioxidant defense components GPX4 and SLC7A11 (encoding xCT) at both the protein and transcript levels, whereas the pro-ferroptotic factor ACSL4 and the inflammatory marker COX2 were significantly upregulated (Figure 5E−G). Therefore, these findings confirm that loss of *SALL2* activates the ferroptosis program in BRCA cells.

To further confirm that ferroptosis mediates the reduction in proliferation observed upon *SALL2* depletion, sh-SALL2 cells were treated with the ferroptosis-specific inhibitor Ferrostatin-1 (Fer-1). Treatment with Fer-1 not only reversed alterations in ferroptosis-related gene expression, restoring GPX4 and SLC7A11 levels while reducing ACSL4 and COX2 expression (Figure 5G−I), but also significantly rescued the impaired proliferation induced by *SALL2* knockdown (Figure 5J). Measurement of intracellular ROS revealed that *SALL2* knockdown exacerbated the accumulation of intracellular ROS compared to the control group (Figure 5K). Consistently, the levels of Fe^2+^ were also increased in sh-SALL2 cells (Figure 5L). Notably, treatment with Fer-1 effectively reversed these phenotypes. These findings indicate that loss of *SALL2* triggers ferroptosis by disrupting iron homeostasis, suppressing the GPX4/SLC7A11 antioxidant axis, and elevating pro-ferroptotic regulators ACSL4 and COX2. Inhibition of ferroptosis effectively counteracts the growth-suppressive effects of *SALL2* deficiency.

Consistent with the *in vitro* results, immunohistochemical analysis of subcutaneous xenograft tumors showed that *SALL2* knockdown reduced *GPX4* and *SLC7A11* expression while increasing *ACSL4* and *COX2* levels compared with control tumors (Figure 5M). These data confirm that *SALL2* modulates BRCA progression by regulating ferroptosis *in vitro* and *in vivo*.

### 2.6. *SALL2* regulates the *ACSL4* promoter region as a transcription factor

**Figure 6.**
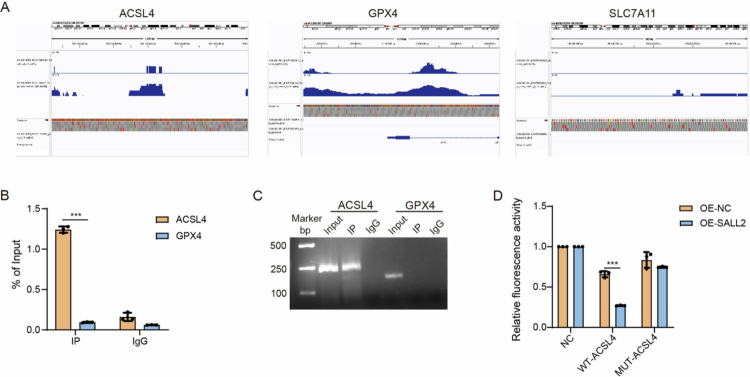
*SALL2* regulates the *ACSL4* promoter region as a transcription factor. A. IGV analysis of SALL2 binding to the promoters of ACSL4, GPX4, and SLC7A11. B,C. Validation of SALL2 binding to the ACSL4 promoter region via ChIP-qPCR and agarose gel electrophoresis. D. Dual luciferase assay verifying *SALL2*'s transcriptional regulatory function on the ACSL4 promoter. (*p* < 0.001,“***”).

To investigate the molecular mechanism by which *SALL2* regulates ferroptosis-associated genes, ChIP-seq datasets for *SALL2* (GSM2824364, ENCLB243VTX) were analyzed. The analysis revealed prominent *SALL2* binding peaks within the promoter regions of *GPX4* and *ACSL4* (Figure 6A). High-confidence peaks (*p* < 1e-5) were identified in the GPX4 promoter region covering −1200 to −200 bp upstream of the transcription start site (TSS), containing a conserved *SALL2* binding motif (5’-[AT] GGG (C/T) GGG [AT]-3’), suggesting that *SALL2* directly binds and transcriptionally activates *GPX4*. Similarly, potent binding signals were observed in the *ACSL4* promoter from -500 to -50 bp upstream of the TSS, with peak intensities comparable to those in GPX4 (reads per million >16) and enriched in GC-box-like sequences (5'-GGG (C/T) GGG-3’), implying potential transcriptional regulation via competitive binding. To experimentally validate these predictions, ChIP-qPCR assays were performed in BRCA cells. The results showed that the SALL2 antibody specifically enriched the *ACSL4* promoter region, whereas enrichment at the *GPX4* promoter was not significantly different from IgG controls (Figure 6B, C), indicating that *SALL2* selectively binds the *ACSL4* promoter in this case. Further analysis using a dual luciferase reporter system revealed that overexpression of *SALL2* (OE-SALL2) significantly suppressed the activity of the wild-type *ACSL4* promoter (Promoter-ACSL4-WT). This repression was completely abolished when the predicted *SALL2* binding site was mutated (Promoter-ACSL4-MUT) (Figure 6D). These results show that *SALL2* directly interacts with the *ACSL4* promoter and functions as a transcriptional repressor, negatively regulating *ACSL4* expression at the transcriptional level.

## 3. Discussion

Breast cancer initiation and progression involve complex dysregulation of various genes and signaling pathways. A comprehensive understanding of the key molecular drivers and control mechanisms underlying BRCA is crucial for advancing diagnostics and treatments. This study highlights *SALL2* as an oncogenic factor in BRCA and elucidates its regulatory network. It shows that *SALL2* levels are controlled by m5C RNA methylation upstream, while *SALL2* promotes tumor progression downstream by suppressing ferroptosis. These understandings suggest that *SALL2* could be an effective target for BRCA therapy.

*SALL2* is a member of the Spalt-like C2H2 zinc finger transcription factor family and is critically involved in embryonic development, particularly in neural and ocular development. However, its role in tumor biology is multifaceted and highly context-dependent. Previous studies have reported that *SALL2* expression is reduced in various malignancies, where it predominantly functions as a tumor suppressor by restricting cell cycle progression. *SALL2* has been shown to directly bind the promoters of cyclin D1 (CCND1) and cyclin E1 (CCNE1), thus repressing their transcription, blocking the G1/S phase transition, and inhibiting cellular proliferation. The inverse correlation between *SALL2* expression and G1/S-associated cyclins observed across multiple cancer types further supports this regulatory model.^40^ ^,^ ^41^ In addition to its anti-proliferative effects, *SALL2* exhibits pro-apoptotic activity, partially resembling p53 function by inducing downstream targets such as *p21* and *BAX.* ^13^ ^,^ ^42^ ^,^ ^43^ The tumor-suppressive activity of *SALL2* is tightly controlled by post-translational regulatory mechanisms. It has been shown that the oncogenic kinase CK2 phosphorylates several conserved residues of SALL2 (S763, T778, S802, and S806), thus facilitating its ubiquitination and subsequent proteasomal degradation. Pharmacological inhibition of CK2 using Silmitasertib restores SALL2 protein levels and induces cancer cell death in a *SALL2*-dependent manner, highlighting *SALL2* as a key mediator of CK2 inhibitor-induced cytotoxicity.^44^ However, oncogenic functions of *SALL2* have been shown in specific cellular contexts. In glioblastoma, *SALL2* contributes to the maintenance of cancer stem cell properties.^45^ ^,^ ^46^ With respect to cell migration, *SALL2* has complex behavior. While *SALL2* deficiency enhances migration in certain cancer cell types, studies in mouse embryonic fibroblasts have revealed that *SALL2* positively regulates cell migration and focal adhesion dynamics by transcriptionally upregulating integrin β1 (ITGB1) and promoting FAK Y397 autophosphorylation.^47^ These findings illustrate the dual, context-specific roles of *SALL2* in cancer biology. *SALL2* suppresses tumor growth by limiting proliferation and promoting programmed cell death. However, it also supports migratory behavior and stem-like features under specific regulatory states and microenvironmental conditions. The functional outcome of *SALL2* activity is therefore determined by cellular context, upstream regulatory modifications such as CK2-mediated phosphorylation, and downstream signaling targets, including cyclins and integrin-associated pathways. This complexity presents both opportunities and challenges for the development of therapeutic strategies targeting *SALL2*. This study reports for the first time that *SALL2* is highly expressed in BRCA and exerts tumor-promoting effects, particularly in HER2-positive and luminal subtypes. These observations expand the current understanding of *SALL2* biology and suggest that *SALL2* may represent a clinically relevant therapeutic target in specific BRCA subgroups.

Given the aberrant overexpression of *SALL2* in BRCA, the upstream regulatory mechanisms responsible for this dysregulation were further investigated. Considering the higher m5C methylation signature associated with *SALL2*, this study was directed toward RNA epigenetic regulation. The m5C methyltransferase NSUN2 was found to be significantly upregulated in BRCA, and its expression showed a strong positive correlation with *SALL2* levels. Mechanistic analyzes revealed that NSUN2 directly catalyzes m5C modification of *SALL2* mRNA through its methyltransferase activity, rather than exerting indirect regulatory effects. Previous studies have reported that the m5C reader protein YBX1 enhances mRNA stability and contributes to BRCA progression.^48^ ^,^ ^49^ Consistent with this, this study shows that YBX1 specifically recognizes the m5C-modified region of mRNA, thus increasing transcript stability. These findings define a coherent upstream regulatory axis comprising “NSUN2 (writer)-m5C-YBX1 (reader)-SALL2 (target),” providing a mechanistic understanding for *SALL2* overexpression at the post-transcriptional level and extending current knowledge regarding NSUN2/YBX1-mediated regulation in cancer.^25^ ^,^ ^27^

NSUN2 is a key enzyme that mediates RNA m5C methylation. Accumulating evidence has shown that NSUN2 is frequently upregulated in diverse malignancies and modulates numerous oncogenic processes, indicating its great potential as a therapeutic target for cancer.^22^ A previous study developed a nanoparticle delivery system for targeted NSUN2 silencing via siRNA, which potently inhibited colorectal cancer metastasis and facilitated T-cell infiltration.^50^ In addition, small molecule NSUN2 inhibitors have been shown to enhance the antitumor effect of doxorubicin and other chemotherapeutics, as well as to confer tumor radiosensitivity.^51^ ^,^ ^52^ Consistent with the results of previous studies, our present study demonstrated significantly elevated expression of *NSUN2* in BRCA. Functional experiments revealed that NSUN2 knockdown inhibited the proliferation and migration of BRCA cells and increased cellular response to paclitaxel. These findings suggest that targeting NSUN2 holds promise as a new strategy for cancer intervention. Genetic silencing or pharmacological inhibition of NSUN2 can efficiently block tumor progression, and combinatorial treatment with conventional chemo-, radio- and immunotherapy can further amplify therapeutic efficacy. Although relevant studies have demonstrated the feasibility of developing NSUN2 as a therapeutic target, its translation into clinical practice still faces numerous challenges: on the one hand, currently reported NSUN2 inhibitors are generally still in the preclinical stage, and their efficacy and safety for clinical use require further validation; on the other hand, NSUN2 expression and the regulatory networks are heterogeneous among different cancers and cancer subtypes, creating a major barrier to individualized screening and precise targeting.

To examine the downstream signaling pathways through which *SALL2* promotes BRCA progression, transcriptomic profiling was performed after *SALL2* silencing. Differentially expressed genes (DEGs) were significantly enriched in ferroptosis-related pathways. This observation was supported by multiple lines of evidence. At the molecular level, *SALL2* depletion significantly downregulated the ferroptosis defense components GPX4 and SLC7A11, and upregulated pro-ferroptotic markers, including ACSL4 and COX2. At the ultrastructural level, TEM revealed characteristic ferroptotic mitochondrial features, such as reduced mitochondrial size and increased membrane density. At the metabolic level, intracellular free iron concentrations were significantly elevated. Functional rescue experiments showed that treatment with the ferroptosis-specific inhibitor Ferrostatin-1 effectively reversed the growth-suppressive effects induced by *SALL2* knockdown. These results collectively indicate that activation of ferroptosis is the primary mechanism by which *SALL2* deficiency inhibits BRCA cell growth. Furthermore, the direct molecular basis of *SALL2*-mediated regulation of ferroptosis was elucidated. Bioinformatic prediction, combined with experimental validation, confirmed that *SALL2* directly binds to the promoter region of *ACSL4*, a key pro-ferroptotic gene, thus repressing its transcriptional activity. This finding establishes a direct transcriptional link between *SALL2* and ferroptosis regulation and provides a novel theoretical basis and therapeutic rationale for targeting ferroptosis pathways in BRCA.

## 4. Conclusion

**Figure 7.**
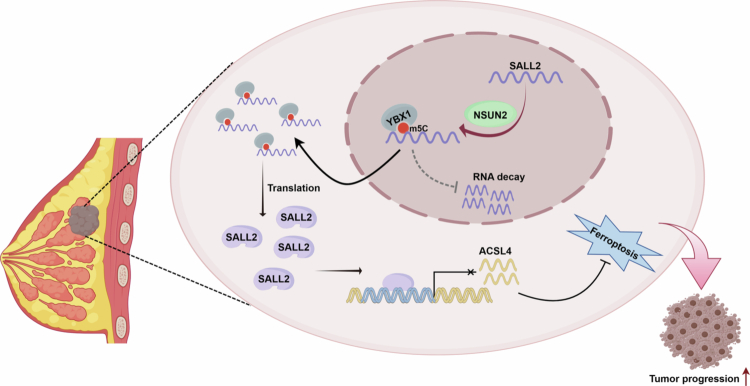
Mechanism Diagram of *NSUN2* mediates *SALL2* m5C methylation to inhibit ferroptosis and promote breast cancer progression. SALL2 undergoes m5C methylation under the regulation of NSUN2/YBX1. This modification enhances the stability of SALL2 mRNA, leading to its upregulation. SALL2 binds to the promoter region of ACSL4, inhibiting its transcriptional activity and causing downregulation of ACSL4 expression. This subsequently suppresses ferroptosis in tumor cells, ultimately promoting the progression of breast cancer tumors.

In conclusion, this study identifies *SALL2* as a key oncogenic driver in breast cancer and systematically elucidates the molecular mechanisms that regulate its expression and function (Figure7). SALL2 is significantly upregulated in breast cancer and is closely associated with poor clinical outcomes. Its suppression significantly impairs tumor cell proliferation, migration, and tumorigenicity. *SALL2* expression is post-transcriptionally regulated by an m5C-dependent regulatory axis where NSUN2 collaborates with YBX1 to mediate RNA methylation and mRNA stability, thereby sustaining SALL2 overexpression. *SALL2* promotes tumor progression by inhibiting ferroptosis through transcriptional repression of the pro-ferroptotic gene ACSL4 and maintenance of the GPX4/SLC7A11 antioxidant system. These findings highlight *SALL2* as a promising prognostic biomarker and a potential therapeutic target for breast cancer treatment.

## 5. Materials and methods

### 5.1. Data acquisition and processing

Transcriptomic and clinical data for breast cancer patients were obtained from The Cancer Genome Atlas (TCGA) database (https://portal.gdc.cancer.gov/) via the TCGAbiolinks R package (v2.25.3). RNA sequencing expression profiles and respective clinicopathological data of BRCA samples were downloaded from the Genomic Data Commons portal. Differential expression analysis of *SALL2* among distinct BRCA subtypes, as well as correlation analysis between *NSUN2* and *SALL2* expression levels, were conducted using the *stats* and *ggplot2* R packages. Survival analysis was performed using the *survival* and *survminer* packages to evaluate the association between *SALL2* expression and patient prognosis, with survival curves generated accordingly.

### 5.2. Cell culture

Human breast cancer cell lines (MDA-MB-231, T47D, and SK-BR-3) were cultured in high-glucose Dulbecco’s Modified Eagle’s Medium (DMEM, Servicebio, G4515) enriched with 10% fetal bovine serum (FBS, Sangon Biotech, E600001) and 1% penicillin-streptomycin (Servicebio, G4003). Human normal mammary epithelial cells (MCF10A) were maintained in breast epithelial cell-specific medium. All cell lines were incubated at 37 °C with 5% CO₂. The SK-BR-3 cell line (Cat. No.: 1101HUM-PUMC000085) was obtained from the Beijing Union Medical College Cell Resource Center. MDA-MB-231 (Cat. No.: SCSP-5043), while T47D (Cat. No.: SCSP-564) and MCF10A (Cat. No.: SCSP-575) cells were procured from the Cell Resource Center of the Shanghai Institute of Life Sciences, Chinese Academy of Sciences. All lines were confirmed by short tandem repeat (STR) profiling, and tests for mycoplasma were negative.

### 5.3. Lentiviral transfection

Lentiviral vectors for gene knockdown and overexpression were constructed and packaged by Shanghai GeneChem Co., Ltd. Knockdown constructs were cloned into the GV493 vector, while overexpression constructs were inserted into the GV492 vector. Cells were seeded in 6-well plates at a density of 2.5 × 10^5^ cells per well and cultured for 16−24 h until reaching 20−30% confluence. Lentiviral transduction was performed according to the titer and multiplicity of infection (MOI). Lentiviral particles were diluted in serum-free medium and mixed with the transfection enhancer HGP (40 μL, GeneChem, REVG005), followed by incubation on ice. The mixture was added to cells and incubated for 14-16 h, after which fresh complete medium was replaced. Stable transfected cell lines were selected using 2 μM puromycin (Beyotime, ST551). Transduction efficiency was monitored using fluorescence microscopy.

### 5.4. Tissue sample collection

Breast cancer tissues and paired normal adjacent tissues were obtained from patients undergoing surgical resection at the Department of Breast Surgery, Liaocheng People’s Hospital. The protocol adhered strictly to the ethical guidelines of the Declaration of Helsinki, and all patients provided written informed consent. Tissue samples were rinsed with sterile saline and immediately stored at −80 °C for further analysis. A portion of the specimens were fixed in 4% paraformaldehyde (PFA, Beyotime, P0099) for 48 h, dehydrated through graded ethanol, and embedded in paraffin for hematoxylin-eosin staining and immunohistochemical analysis.

### 5.5. Real-time quantitative PCR (RT-qPCR)

Total RNA was extracted and reverse-transcribed into cDNA using Vazyme reverse transcription kits (R323). Quantitative real-time PCR was performed with the Vazyme qPCR kit (Q712) according to the manufacturer’s instructions. Primer sequences were obtained from PrimerBank and synthesized by Sangon Biotech.

### 5.6. Western blotting

Cells were lysed in RIPA buffer (Beyotime, P0013B) containing 1% PMSF (Beyotime, ST506) for total protein extraction. Protein concentrations were determined using a BCA protein assay kit (Beyotime, P0012). Proteins were denatured in 5X SDS loading buffer (Beyotime, P0015), separated by SDS-PAGE, and transferred onto 0.2 μm PVDF membranes (Millipore, ISEQ00010). Membranes were blocked with 5% non-fat milk for 1.5 h and incubated with primary antibodies at 4 °C overnight. After washing with TBST (Servicebio, G2150), membranes were incubated with HRP–conjugated secondary antibodies at room temperature for 1.5 h. Protein signals were visualized using enhanced chemiluminescence reagents (Millipore, WBULP). The antibodies used are as follows: NSUN2 (1:5000, Proteintech, 20854-1-AP), SALL2 (1:1000, Proteintech, 12679-1-AP), GPX4 (1:1000, Abcam, ab125066), ACSL4 (1:1000, Abcam, ab205199), COX2 (1:1000, CST, #12282), SLC7A11 (1:1000, Abcam, ab307601), GAPDH (1:10000, Abcam, ab181602), HRP-labeled goat anti-rabbit IgG (H + L) (1:1000, Beyotime, A0208), HRP-labeled goat anti-mouse IgG (H + L) (1:1000, Beyotime, A0216).

### 5.7. Hematoxylin-eosin staining (HE)

Paraffin-embedded tissue sections (5 μm thickness) were mounted on glass slides and kept at 60 °C for 2 h. The sections were deparaffinized in xylene and rehydrated through a graded ethanol series to 80% ethanol. Hematoxylin (Beyotime, C0105S) staining was performed for 30–60 s, followed by differentiation in 0.3% HCL and bluing in 1% ammonium hydroxide. Sections were then counterstained with water-soluble eosin (Beyotime, C0105S). Each staining step was followed by rinsing under running tap water for 1–2 min. Finally, the sections were dehydrated through graded ethanol, cleared in xylene, mounted with neutral resin (Beyotime, C0173), and examined under a light microscope to evaluate histological morphology and staining quality. Ethanol, xylene, HCl, and ammonium hydroxide were purchased from Yantai Shuangshuang Chemical Co., Ltd., AR.

### 5.8. Immunohistochemical analysis (IHC)

Paraffin-embedded tissue sections were kept at 60 °C to remove residual paraffin, followed by immunohistochemical staining performed using the BOND-MAX automated staining system (M495343). Briefly, sections were deparaffinized in xylene and rehydrated through a graded ethanol series to 80% ethanol. Antigen retrieval was carried out using citrate buffer or EDTA buffer for 20 min, followed by endogenous peroxidase blocking for 5 min. Sections were incubated with primary antibodies for 60 min and washed, then incubated with HRP–conjugated goat anti-mouse or goat anti-rabbit IgG polymer for 8 min. Signal was detected using DAB chromogen for 10 min, and nuclei were counterstained with hematoxylin for 8 min. The H-scores of the IHC images were determined using ImageJ.

The primary antibodies used in this study included NSUN2 (1:1000, Proteintech, 20854-1-AP), SALL2 (1:100, Invitrogen, PA5-52065), GPX4 (1:500, Abcam, ab125066), ACSL4 (1:300, Abcam, ab205199), COX2 (1:200, CST, #12282), SLC7A11 (1:50, Abcam, ab307601), and Ki-67 (1:200, Abcam, ab16667).

### 5.9. Cell viability and proliferation assay

Cell viability was examined using the Cell Counting Kit-8 (CCK-8) (Beyotime, C0039) assay. Cells (5 × 10^4^ cells/mL) were seeded into 96-well plates and incubated under standard culture conditions. At respective time points (0, 24, 48, and 72 h), 10 μL CCK-8 reagent was added and incubated for 2 h. Absorbance was measured at 450 nm using a microplate reader. For ferroptosis inhibition experiments, cells were treated with 2 μM Ferrostatin-1 (MCE, HY-100579).

### 5.10 Colony formation assay

Cells (1,000 cells/well) were seeded into 6-well plates and cultured at 37 °C with 5% CO₂, with fresh medium replaced at regular intervals. When visible colonies comprising more than 50 cells had formed, cultures were gently washed with PBS (Servicebio, G4202), fixed, and stained with 5% crystal violet solution (Beyotime, Y268091). Excess dye was removed by washing with distilled water. Colonies were observed and quantitatively analyzed using ImageJ software.

### 5.11. Wound healing assay

Cells were evenly seeded (1 × 10^6^ cells/well) in 6-well plates. Once they reached over 90% confluence, a linear scratch was induced in the cell monolayer with a sterile pipette tip. Detached cells were gently washed away with PBS, and the culture medium was replaced with medium containing 2% FBS. Images of the wounded areas were acquired at 0, 24, and 48 h using a light microscope, with the same field of view captured at each time point. Wound areas were measured using ImageJ, and cell migration rates were calculated based on these measurements.

### 5.12. Subcutaneous tumor formation assay

Five-week-old female BALB/c nude mice (Henan Skebes Biotechnology Co., Ltd.), weighing approximately 20 g (10 mice in total), were housed under controlled conditions (26 °C, 40-50% humidity) with free access to food and water under a 12 h light/12 h dark cycle. After a 3-day acclimation period, the mice were randomly assigned to two groups of five mice each using the Excel randomization. T47D cells, either expressing control shRNA (sh-NC) or SALL2-targeting shRNA (sh-SALL2), were injected subcutaneously into the flanks of mice. Tumor growth and overall health were monitored regularly, with tumor volume and body weight measured throughout the experimental period (tumor diameters < 2 cm); mice that did not develop tumors for reasons unrelated to the experiment were removed from the study. At the end of the study, the mice were euthanized by cervical dislocation after anesthesia with isoflurane (RWD, R510), and the tumors were excised, weighed, and fixed in PFA for immunohistochemistry. This study strictly adhered to the ARRIVE guidelines.

### 5.13. RNA stability assay

RNA stability was evaluated using an actinomycin D-mediated transcriptional inhibition assay after NSUN2 knockdown. Cells were seeded (3 × 10^4^ cells/well) into 6-well plates. After 24 h, cells from one well were harvested for RNA extraction and designated the 0 h time point. The remaining wells were treated with medium containing actinomycin D (10 μg/mL, MCE, HY-17559), and cells were collected at 1, 2, and 4 h post-treatment. Total RNA was extracted, and quantitative RT-qPCR was performed. ΔCt values were calculated relative to the 0 h group, and relative mRNA abundance at each time point was determined using the formula 2^^−^ΔCt^.

### 5.14. RNA dot blot

Total RNA was extracted from control and NSUN2 knockdown cells, and RNA concentrations were quantified. Equal amounts of RNA (1,200 ng, 600 ng, and 300 ng) were prepared in DEPC-treated water (Beyotime, R0021). Samples were denatured at 95 °C for 3 min, immediately cooled on ice, and briefly centrifuged. RNA samples were then uniformly spotted onto NC membranes (Millipore, HATF08130) and incubated at 37 °C for 30 min to allow cross-linking. Membranes were blocked and incubated overnight at 4 °C with an anti-m5C antibody (1:2500, Proteintech, 68301-1-Ig), followed by incubation with the appropriate secondary antibody and signal development. Membranes were then stained with 10% methylene blue (Sigma, M9140) and imaged after removal of excess dye with distilled water to serve as a loading reference.

### 5.15. MeRIP-qPCR

Methylated RNA immunoprecipitation followed by quantitative PCR (MeRIP-qPCR) was performed using an m5C Methylated RNA Immunoprecipitation Kit (Bersin Bio, Bes5204) in accordance with the manufacturer’s protocol. Total cellular RNA was isolated, and RNA integrity and concentration were examined before fragmentation (100 nucleotides in length). Fragmented RNA was divided into immunoprecipitation (IP) and input fractions. The IP samples were incubated with an anti-m5C antibody (5 μg, Proteintech, 68301-1-Ig), and antibody–RNA complexes were captured using Protein A/G magnetic beads. After immunoprecipitation, RNA was purified and analyzed via RT-qPCR. Enrichment levels were calculated relative to the corresponding input samples.

### 5.16. GSH assay

The assay was performed using the GSH and GSSG assay kit (Beyotime, S0053). Cells were collected by centrifugation and processed according to the instructions of the kit. One portion of cells was used for measurement of total glutathione, while the other portion was treated with GSH-removal working solution for GSSG determination. The total glutathione concentration, GSH concentration, and GSH/GSSG ratio were calculated for each sample based on the standard curve.

### 5.17. Cellular chemosensitivity assay

Cells in the logarithmic growth phase were seeded in 96-well plates and cultured for 24 h, after which the medium was replaced with medium containing paclitaxel (albumin-bound) solution (CSPC Ouyi Pharmaceutical Co., Ltd.) to yield final paclitaxel concentrations of 0, 0.01, 0.1, 1, and 10 μM. After further culture for 48 h, cell viability was assessed using a CCK-8 assay kit.

### 5.18. RNA immunoprecipitation (RIP)

RNA immunoprecipitation (RIP) assays were conducted using an RNA Immunoprecipitation Kit (Bersin Bio, Bes5101). Approximately 1 × 10^7^ cells were harvested, lysed, and treated to remove genomic DNA. Cell lysates were incubated with specific antibodies overnight at 4 °C under gentle rotation. Protein A/G magnetic beads were added to capture immune complexes. After washing, RNA was eluted and purified. The enrichment of target RNAs was evaluated by quantitative PCR and agarose gel electrophoresis. Antibodies against YBX1 (5 μg, Proteintech, 20339-1-AP), ACSL4 (5 μg, Abcam, ab205199), and GPX4 (5 μg, Abcam, ab125066) were used.

### 5.19. Sequencing of mRNA

Total RNA was extracted from cultured cells and analyzed via high-throughput RNA sequencing by Shanghai Bohao Biotechnology Co., Ltd. RNA samples were purified and quality controlled before mRNA enrichment and fragmentation. Sequencing libraries were constructed following standard protocols and sequenced on the Illumina NovaSeq 6000 platform. Bioinformatic analysis was performed to obtain gene expression profiles.

### 5.20. Transmission electron microscope (TEM) scanning

Cells were collected at a confluence not exceeding 70%, and approximately 1 × 10^7^ cells were harvested by centrifugation. Cell pellets were resuspended in electron microscopy fixative (1 mL, Servicebio, G1102) and fixed at room temperature in the dark for 30 min, followed by storage at 4 °C. Ultrastructural analysis was conducted by Servicebio Biotechnology Co., Ltd. using TEM, with particular focus on mitochondrial morphology.

### 5.21. Iron ion concentration detection

Intracellular iron ion levels were quantified using an iron assay kit (Applygen Technologies, E1042). Approximately 2 × 10^6^ cells were collected, lysed, and centrifuged to obtain the supernatant. Protein concentrations were determined for normalization. A buffer solution and 4.5% potassium permanganate solution were mixed at a 1:1 ratio to generate the reaction mixture. The mixture was combined with 100 μL of sample or standard solution and incubated at 60 °C for 1 h. After cooling, the iron detection reagent (30 μL) was added and incubated at room temperature for 30 min. Samples were centrifuged at 12,000 rpm for 5 min, and the absorbance of the supernatant was measured at 550 nm. Iron concentrations were calculated using a standard curve and normalized to total protein content.

### 5.22. ROS measurement

Cells were evenly seeded in 6-well plates and cultured overnight. The cells were then washed and the DHE probe (Bestbio, BB-47051) was added and incubated for 25 min at 37 °C in the dark. After washing to remove the unbound probe, Hoechst 33342 (Beyotime, C1022) was added and incubated for 10 min at room temperature in the dark. After further washing, the cells were evaluated and imaged under fluorescence microscopy.

### 5.23. Fe^2+^ measurement

Cells seeded in 6-well plates were treated with Hoechst 33342 (Beyotime, C1022) and incubated for 10 min at room temperature in the dark. The cells were then washed, followed by addition of the Fe^2+^ detection probe (MeilunBio, MA0647), incubation for 35 min at 37 °C in the dark, and observation and imaging under fluorescence microscopy.

### 5.24. ChIP-qPCR

Chromatin immunoprecipitation followed by quantitative PCR (ChIP–qPCR) was carried out using a ChIP Assay Kit (Beyotime, P2078). When cells reached approximately 80% confluence, formaldehyde (Yantai Shuangshuang Chemical Co., Ltd., AR) was added directly to the culture medium to a final concentration of 1% to cross-link protein–DNA complexes. After incubation at 37 °C for 10 min, cells were harvested, lysed, and sonicated to shear chromatin. The resulting lysates were centrifuged, and the supernatants were diluted with ChIP dilution (2 mL) buffer containing 1 mM PMSF. Immunoprecipitation was performed, and enriched DNA fragments were purified and analyzed by qPCR to examine target gene promoter occupancy.

### 5.25. Dual luciferase assay

Dual-luciferase reporter assays were conducted using plasmids constructed by Shanghai GeneChem Co., Ltd. Cells were seeded (8 × 10^4^ cells/well) into 24-well plates and transfected when confluence reached approximately 60%. Transfection complexes were prepared by mixing 0.5 μg plasmid DNA with 2 μL X-tremeGENE HP (Merck, 6366236001) transfection reagent in DMEM (100 μL) and incubating at room temperature for 20 min. A total of 200 μL of medium was retained in the plate wells, and the above mixture was added, and incubated for 5–6 h, followed by replacement with complete medium (500 μL). After 24-48 h, cells were harvested for luciferase activity measurement using a dual-luciferase assay kit (E1910, Promega). Firefly luciferase activity was measured first, followed by Renilla luciferase activity after the addition of Stop & Glo reagent. Relative luciferase activity was calculated as the ratio of firefly to Renilla luminescence.

### 5.26. Statistical analysis

Data were statistically examined using SPSS software (v27.0.1). Graphical representations were generated using GraphPad Prism (v8.0.2). Data are presented as mean ± standard deviation. Two groups were compared using Student’s *t*-test, while multiple groups were compared by ANOVA. A *p-*value < 0.05 was considered statistically significant. All experiments were repeated at least three times.

## Supplementary Material

Supplementary Material Author_Checklist.pdf

## Data Availability

The data that support the findings of this study are openly available in Zenodo at https://doi.org/10.5281/zenodo.20554592.
